# Supplementary material for: Cholangiocytes contribute to hepatocyte regeneration after partial liver injury during growth spurt in zebrafish
Source: Nat Commun. 2025 Jun 6;16:5260. doi: 10.1038/s41467-025-60334-y (PMC12144294; doi:10.1038/s41467-025-60334-y)
Supplement: Supplementary file 1 — Supplementary Information [file 41467_2025_60334_MOESM1_ESM.pdf]

SUPPLEMENTARY INFORMATION

Cholangiocytes contribute to hepatocyte regeneration after partial liver injury during growth spurt in zebrafish.

Sema Elif Eski[1], Jiarui Mi[2,3], Macarena Pozo-Morales[1,#], Gabriel Garnik Hovhannisyan[4], Camille Perazzolo[1], Rita Manco[5], Imane Ez-Zammoury[6], Dev Barbhaya[7], Anne Lefort[1], Frédérick Libert[1], Federico Marini[8,9], Esteban N. Gurzov[4], Olov Andersson[2,10], Sumeet Pal Singh[1,11,*]

[1] Laboratory of Regeneration and Stress Biology, Institut de Recherche Interdisciplinaire en Biologie Humaine et Moléculaire (IRIBHM-Jacques E. Dumont), Université libre de Bruxelles, Brussels, Belgium.

[2] Department of Cell and Molecular Biology, Karolinska Institutet, 17177 Stockholm, Sweden

[3] Department of Gastroenterology, Sir Run Run Shaw Hospital, School of Medicine, Zhejiang University, China

[4] Signal Transduction and Metabolism Laboratory, Université libre de Bruxelles, Anderlecht, 1070 Brussels-Capital Region, Belgium

[5] Laboratory of Hepato-gastroenterology, Institut de Recherche Expérimentale et Clinique, Université Catholique de Louvain, Brussels, Belgium

[6] Faculty of Pharmacy and Biomedical Sciences, Université Catholique de Louvain, Brussels, Belgium

[7] Indian Institute of Technology Kanpur (IIT-Kanpur), Kanpur, India

[8] Institute of Medical Biostatistics, Epidemiology and Informatics (IMBEI), University Medical Center of the Johannes Gutenberg University Mainz, Mainz, Germany

[9] Research Center for Immunotherapy (FZI), Mainz, Germany

[10] Department of Medical Cell Biology, Uppsala University, Biomedical Centre, Uppsala, Sweden

[11] Department of Life Sciences, School of Natural Sciences, Shiv Nadar Institution of Eminence, Delhi, India

[#] Present Address: Molecular Oncology, Spanish National Cancer Research Centre (CNIO), Madrid, Spain

* Corresponding author**:** sumeet.pal.singh@ulb.be

Table of Contents

SUPPLEMENTARY FIGURE 1: RAPID INCREASE OF HEPATOCYTE POPULATION IN LATE LARVAL STAGE ZEBRAFISH................................4

SUPPLEMENTARY FIGURE 2: HIGHLY EFFICIENT CRE/LOX RECOMBINATION IN *TG(FABP10A:CELLCOUSIN)* LIVERS............................6

SUPPLEMENTARY FIGURE 3: CHOLANGIOCYTES ARE THE SOURCE OF *DE NOVO* HEPATOCYTES AFTER PARTIAL ABLATION.....................7

SUPPLEMENTARY FIGURE 4: MORPHOLOGICAL RECOVERY OF THE LIVER AFTER PHX...9

SUPPLEMENTARY FIGURE 5: SINGLE-CELL RNA-SEQUENCING REVEALS TRANSCRIPTOME OF *DE NOVO* HEPATOCYTES FOLLOWING ACUTE INJURY.. 11

SUPPLEMENTARY FIGURE 6: TRAJECTORY ANALYSIS ON CHOLANGIOCYTE SUBPOPULATIONS.. 13

SUPPLEMENTARY FIGURE 7: INHIBITION OF NOTCH SIGNALLING PATHWAY INDUCES SPONTANEOUS TRANSDIFFERENTIATION EVEN IN THE ABSENCE OF ABLATION.. 14

SUPPLEMENTARY FIGURE 8: HER2+ CELL LINEAGE MAINLY CONTAINS CHOLANGIOCYTES...16

SUPPLEMENTARY FIGURE 9: HER9+ CELL LINEAGE CONTAINS CHOLANGIOCYTES AND HAND2+ CELLS, BUT NOT HEPATOCYTES.......... 18

SUPPLEMENTARY FIGURE 10: LINEAGE TRACING STRATEGY TO DEMONSTRATE THE CONTRIBUTION OF HER9+ CELLS LINEAGE TO *DE NOVO* HEPATOCYTES.. 19

SUPPLEMENTARY FIGURE 11: COMPARATIVE TRANSCRIPTOMIC ANALYSIS OF CHOLANGIOCYTE SUBCLUSTERS FROM LARVAL AND ADULT ZEBRAFISH LIVERS..21

SUPPLEMENTARY FIGURE 12: MTORC1 SIGNALLING DIFFERS BETWEEN EARLY AND LATE LARVAL STAGES................................... 23

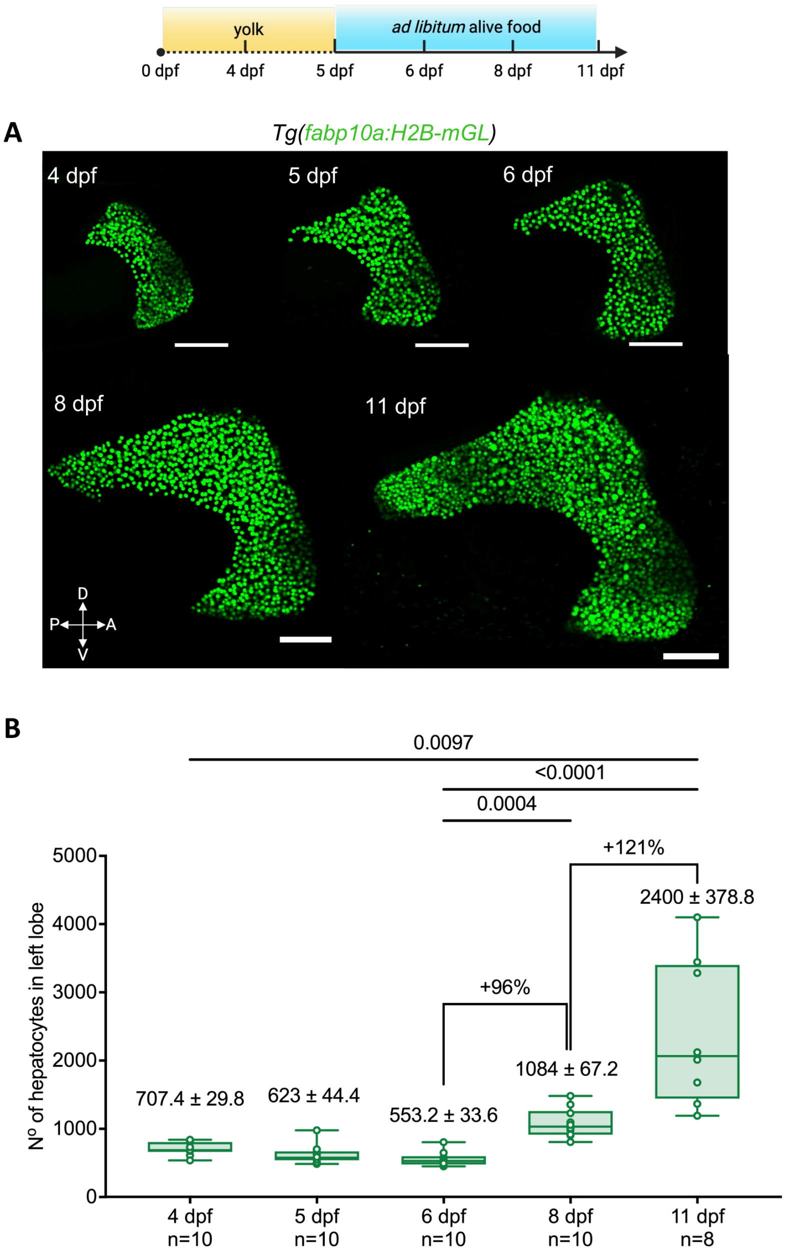

Supplementary Figure 1: Rapid increase of hepatocyte population in late larval stage zebrafish.

(A) Until 5 dpf, zebrafish larvae obtain their nutrients from the yolk. From 5 dpf onwards, they are provided with ad libitum live food, specifically rotifers. Live confocal images of the left lobe of *Tg(fabp10a:H2B-mGreenLantern)* zebrafish liver at 4, 5, 6, 8 and 11 dpf. Scale bars: 100 μm. **(B)** Growth curve was assessed by quantifying the number of hepatocytes in the left lobe. Data are represented as min-to-max box plots

with number of animals listed below. Each dot represents one animal; mean ± SEM is indicated above each box. Quantification revealed a significant increase in hepatocyte numbers, with a 96% increase observed between 6 and 8 dpf, and a 121% increase between 8 and 11 dpf (Kruskal-Wallis test). These findings underscore the rapid proliferation of hepatocytes during this critical growth period.

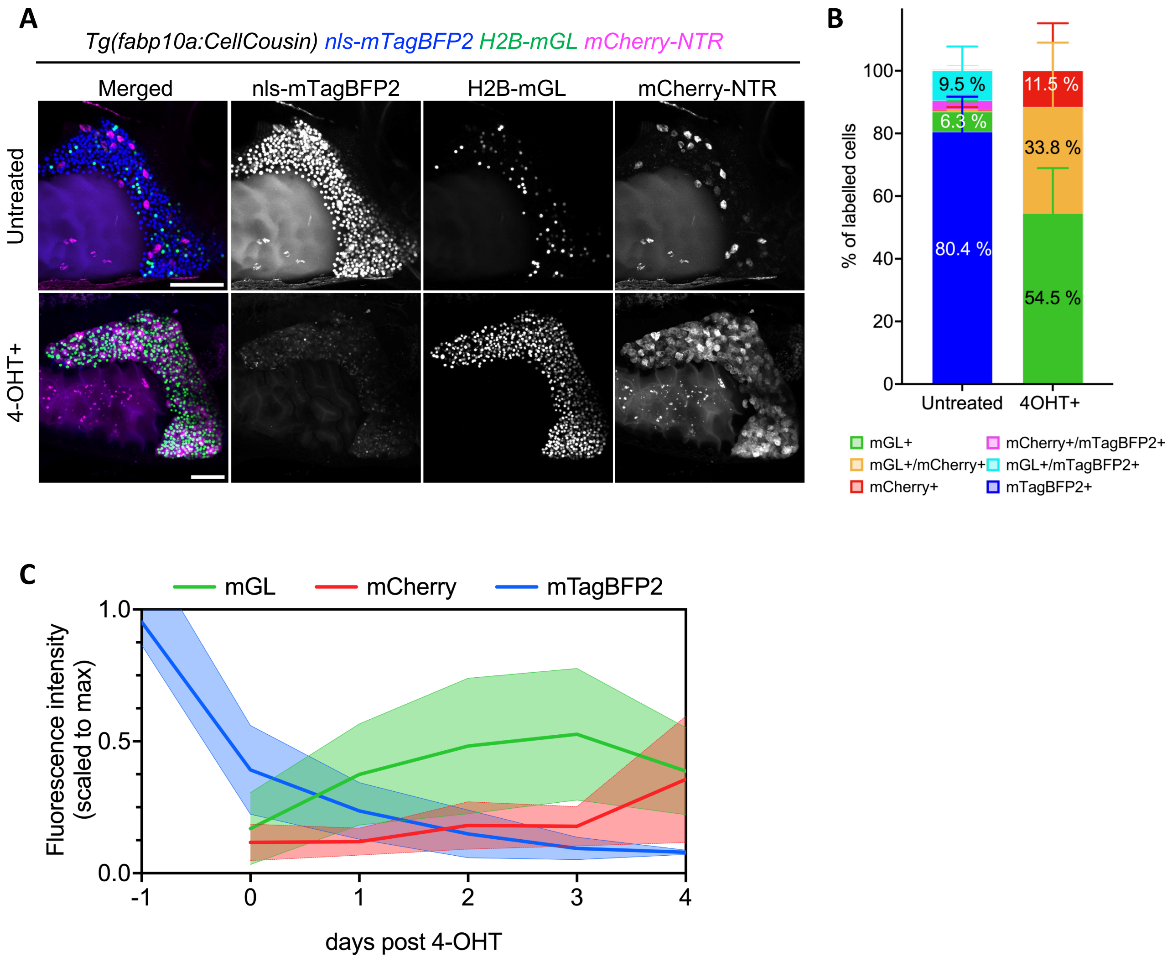

Supplementary Figure 2: Highly efficient Cre/Lox recombination in *Tg(fabp10a:CellCousin)* livers.

(A) Live confocal imaging of liver in 9 dpf zebrafish larvae untreated and 4-OHT-treated. In untreated animals, presence of H2B-mGL+ and mCherry+ hepatocytes show the background recombination due to leaky CreER^{T2} recombination driven by the *fabp10a* promoter. 4-OHT-treatment lead to the switching of nls-mTagBFP2 fluorescence label to H2B-mGL (nuclear green) or mCherry (cytoplasmic magenta). **(B)** Mean ± SD of the percentage of hepatocyte labelling in untreated (n=9 animals) and after 4-OHT treatment (n=5 animals). Scale bars: 100 μm (A). **(C)** Line plot showing the changes in fluorescence after 4-OHT treatment.

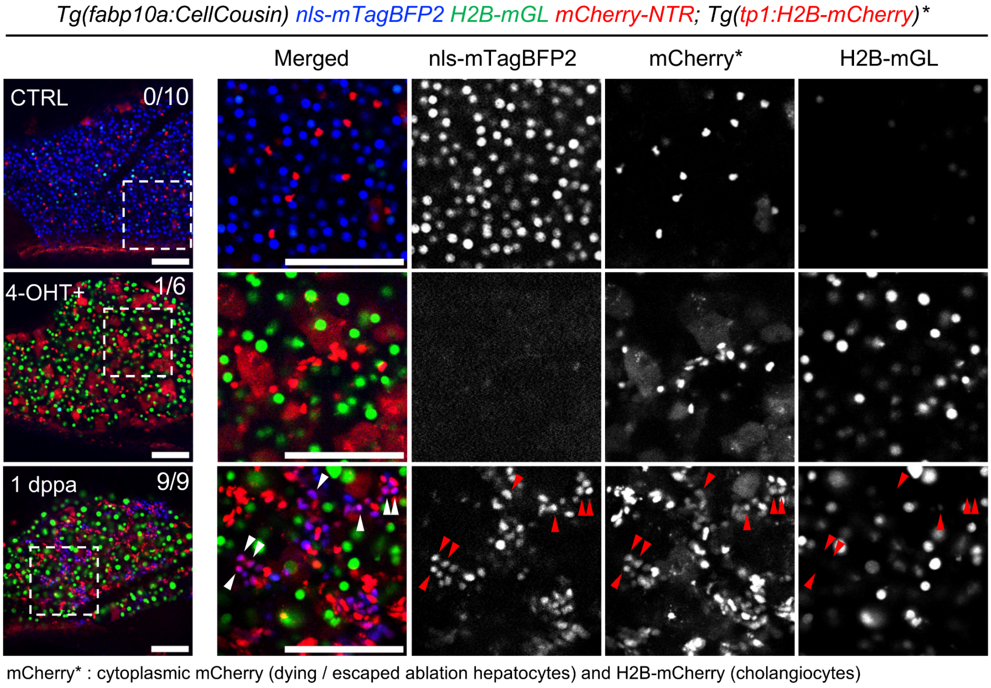

Supplementary Figure 3: Cholangiocytes are the source of *de novo* hepatocytes after partial ablation.

Confocal images of livers from *Tg(fabp10a:CellCousin); Tg(tp1:H2B-mCherry)* in 13 dpf untreated, 4-OHT-treated and 1 dppa zebrafish larvae using *in vivo* live imaging. nls-mTagBFP2+/H2B-mCherry+ cells were observed in 0 / 10 livers in untreated animals, in 1 / 6 livers in 4-OHT treated animals, and in 9 / 9 livers at 1 dppa after MTZ administration. Scale bars: 100 μm (B).

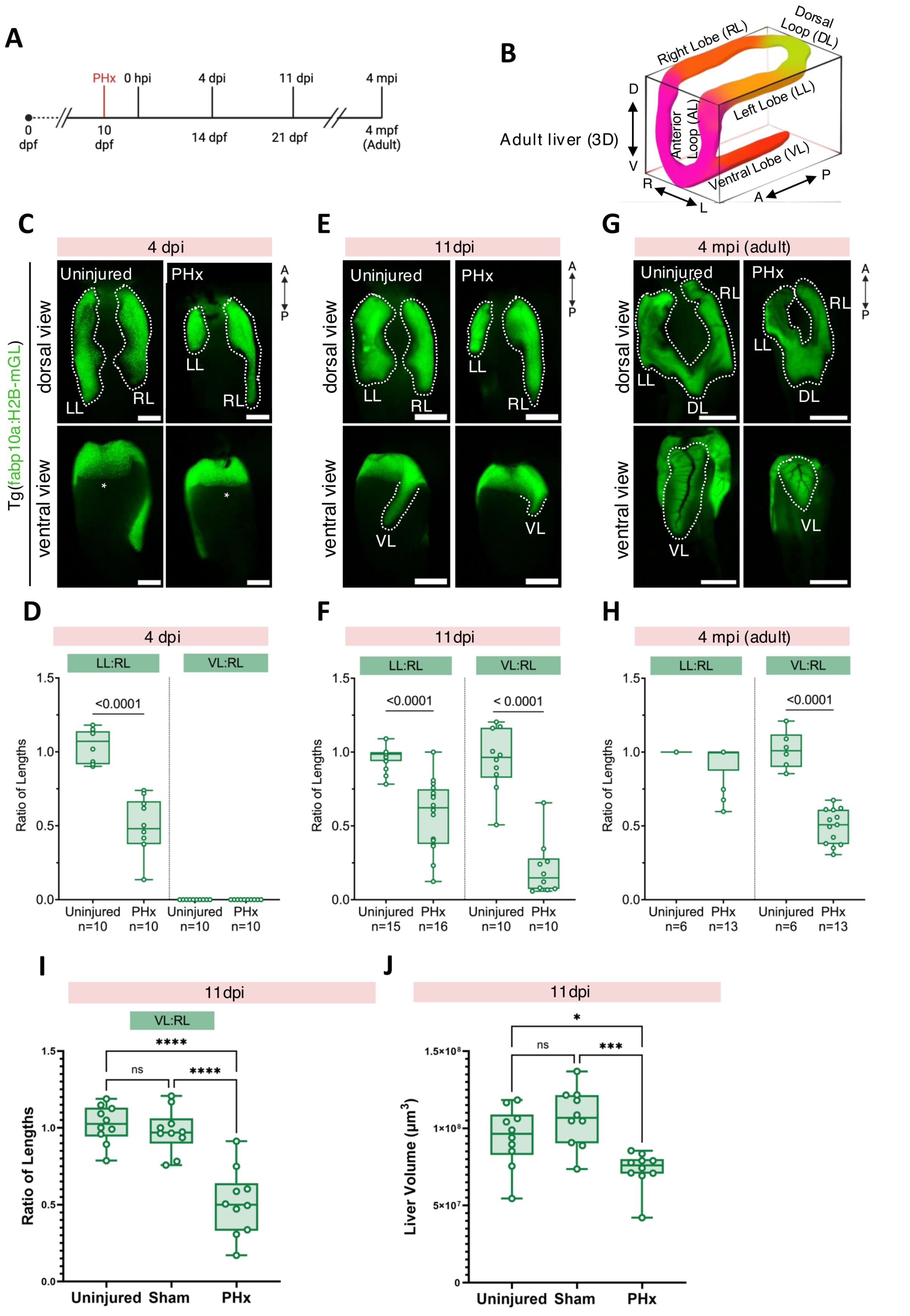
A
PHx
0 hpi
4 dpi
11 dpi
4 mpi
0 dpf
10 dpf
14 dpf
21 dpf
4 mpf (Adult)
B
Adult liver (3D)
Right Lobe (RL)
Dorsal Loop (DL)
Left Lobe (LL)
Anterior Loop (AL)
Ventral Lobe (VL)
D
V
R
L
A
P
C
4 dpi
Tg(fabp10a:H2B-mGL)
dorsal view
ventral view
Uninjured
PHx
LL
RL
E
11dpi
VL
G
4 mpi (adult)
DL
D
LL:RL
VL:RL
Ratio of Lengths
<0.0001
Uninjured n=10
PHx n=10
F
< 0.0001
Uninjured n=15
PHx n=16
H
Uninjured n=6
PHx n=13
I
****
ns
Uninjured
Sham
J
*
***
Liver Volume (µm³)

Supplementary Figure 4: Morphological recovery of the liver after PHx.

(A) Experimental strategy for assessing regeneration after PHx. **(B)** Schematic illustrating liver lobes of an adult zebrafish. **(C, E, G)** Whole-mount images of liver (green) with dorsal (above) and ventral (below) views (left lobe, LL; right lobe, RL; ventral lobe, VL) at 4 dpi (asterisk: missing VL) (E), at 11 dpi (G), and at 4 mpi (J). **(D, F, H)** Min-to-max box plots, with number of animals listed below, showing the quantifications of the ratio of the lengths of LL to RL and VL to RL at 4 dpi (n=10 animals, each) (D), at 11 dpi (uninjured n=15 animals, PHx n=16 animals) (F), and at 4 mpi (uninjured n=6 animals, PHx n=13 animals) (H) (Mann-Whitney test). Scale bars: 200 μm (C), 500 μm (E), 2 mm (G). **(I)** Min-to-max box plots showing quantification of the ratio of the lengths of VL to RL at 11 dpi (n=10 animals, each) comparing uninjured with sham surgery and partial hepatectomy (one-way ANOVA, followed by Tukey's multiple comparison test). **(J)** Min-to-max box plots showing quantification of the liver volume at 11 dpi (n=10 animals, each) comparing uninjured with sham surgery and partial hepatectomy (Kruskal-Wallis Test, followed by Dunn's multiple comparison test). (**** p-value<0.0001; *** p-value=0.009; * p-value=0.0475).

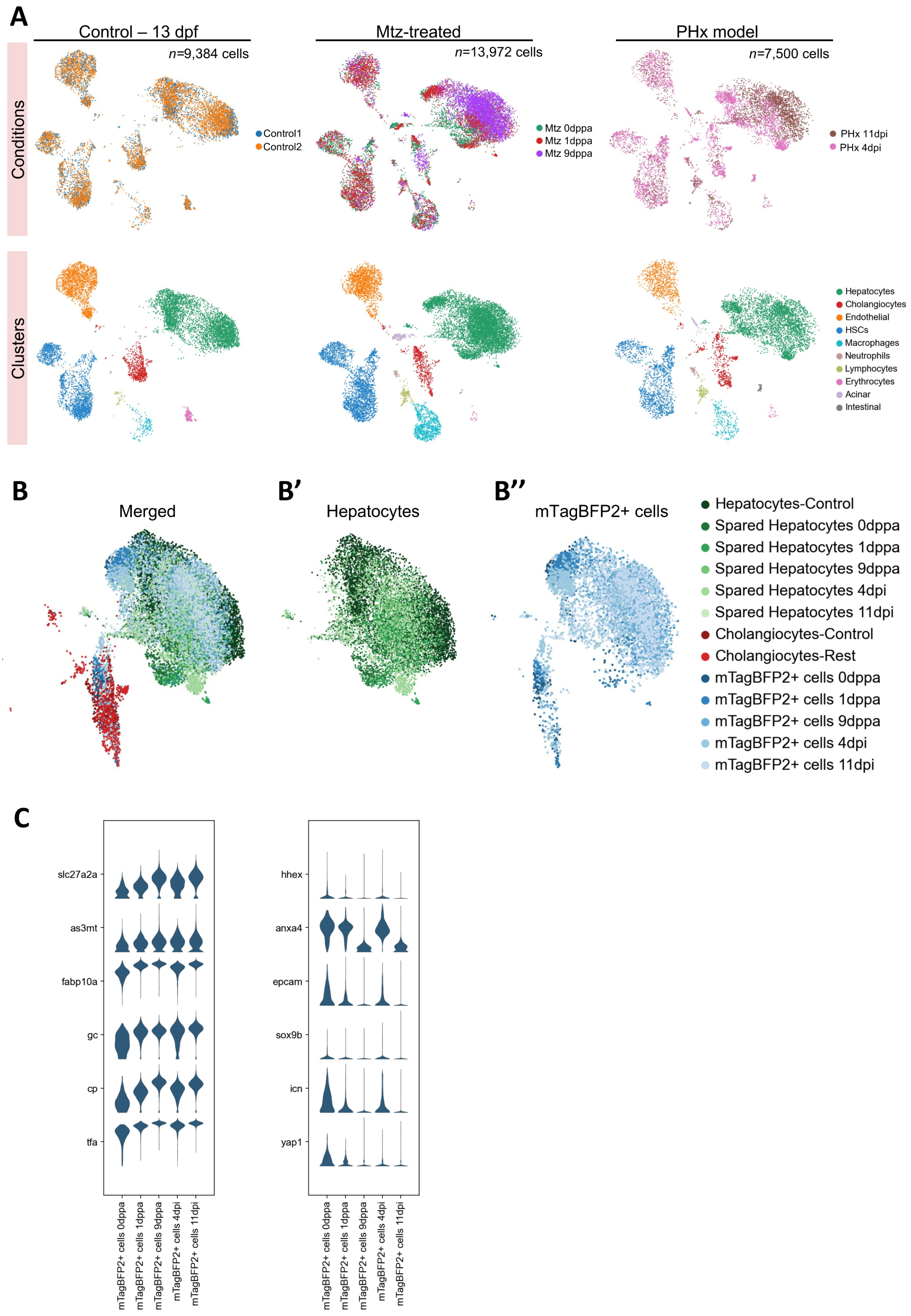
A
Control – 13 dpf
n=9,384 cells
Mtz-treated
n=13,972 cells
PHx model
n=7,500 cells
Conditions
Clusters
Control1
Control2
Mtz 0dppa
Mtz 1dppa
Mtz 9dppa
PHx 11dpi
PHx 4dpi
Hepatocytes
Cholangiocytes
Endothelial
HSCs
Macrophages
Neutrophils
Lymphocytes
Erythrocytes
Acinar
Intestinal
B
Merged
B'
Hepatocytes
B''
mTagBFP2+ cells
Hepatocytes-Control
Spared Hepatocytes 0dppa
Spared Hepatocytes 1dppa
Spared Hepatocytes 9dppa
Spared Hepatocytes 4dpi
Spared Hepatocytes 11dpi
Cholangiocytes-Control
Cholangiocytes-Rest
mTagBFP2+ cells 0dppa
mTagBFP2+ cells 1dppa
mTagBFP2+ cells 9dppa
mTagBFP2+ cells 4dpi
mTagBFP2+ cells 11dpi
C
slc27a2a
as3mt
fabp10a
gc
cp
tfa
hhex
anxa4
epcam
sox9b
icn
yap1
mTagBFP2+ cells 0dppa
mTagBFP2+ cells 1dppa
mTagBFP2+ cells 9dppa
mTagBFP2+ cells 4dpi
mTagBFP2+ cells 11dpi

Supplementary Figure 5: Single-cell RNA-sequencing reveals transcriptome of *de novo* hepatocytes following acute injury.

(A) UMAP visualization of zebrafish larval liver cells per condition: uninjured control (left, *n*=9,384 cells), after Mtz treatment (middle, *n*=13,972 cells) and after partial hepatectomy (PHx, *n*=7,500 cells). The top panels show the cells per condition, while the bottom panels display the clusters. **(B)** UMAP highlighting clusters of interest: control cholangiocytes (dark red), cholangiocytes-rest (from injured livers, red), control hepatocytes (dark green) and spared hepatocytes (greens) and mTagBFP2+ cells (blues). Subsets of hepatocyte and mTagBFP2+ cell clusters at different stages after injury are represented with shades of green and blue, respectively. **(C)** Violin plots showing normalized gene expression of hepatocyte-specific markers (left) and cholangiocyte markers (right) in mTagBFP2+ cells across different timepoints in injured conditions.

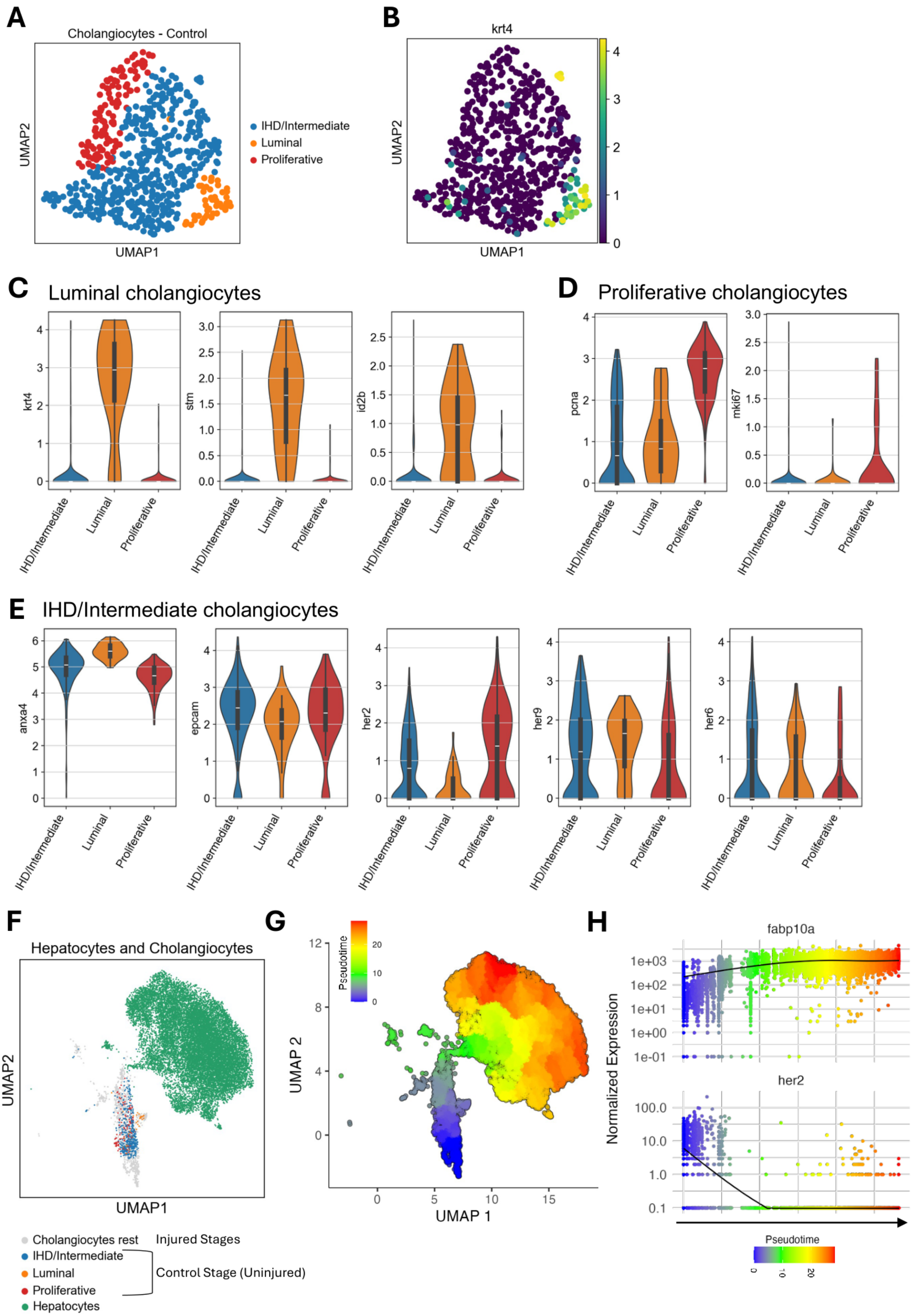
A
Cholangiocytes - Control
UMAP2
UMAP1
IHD/Intermediate
Luminal
Proliferative
B
krt4
UMAP2
UMAP1
C
Luminal cholangiocytes
krt4
stm
id2b
IHD/Intermediate
Luminal
Proliferative
D
Proliferative cholangiocytes
pcna
mki67
E
IHD/Intermediate cholangiocytes
anxa4
epcam
her2
her9
her6
F
Hepatocytes and Cholangiocytes
UMAP2
UMAP1
Cholangiocytes rest
Injured Stages
IHD/Intermediate
Luminal
Control Stage (Uninjured)
Proliferative
Hepatocytes
G
Pseudotime
UMAP 2
UMAP 1
H
fabp10a
her2
Normalized Expression
Pseudotime

Supplementary Figure 6: Trajectory analysis on cholangiocyte subpopulations. (A) UMAP visualization of control cholangiocytes, identifying three distinct subpopulations: luminal cholangiocytes (*n*=44 cells), intrahepatic (IHD)/intermediate cholangiocytes (*n*=448 cells), and a proliferative population enriched in cell-cyle markers (*n*=117 cells). **(B)** Normalized gene expression of krt4, a marker for luminal cholangiocytes, shown on the UMAP. **(C-E)** Violin plots showing the normalized expression of characteristic marker genes for each subpopulation: (C) luminal cholangiocytes, (D) proliferative cholangiocytes, and (E) IHD/intermediate cholangiocytes. **(F)** UMAP visualization of integrated single-cell data from both uninjured and injured livers, including cholangiocyte subclusters from controls (colored) and “cholangiocytes rest” (grey) from injured samples. Hepatocytes are shown in green. **(G)** Pseudotime trajectory inferred using Monocle, showing progression from cholangiocytes to hepatocytes. Analysis was performed on the full integrated dataset, including both uninjured and injured samples, to capture transitional states absent from control livers. **(H)** Trendline plot showing the dynamic expression of fabp10a and her2 along the pseudotime path.

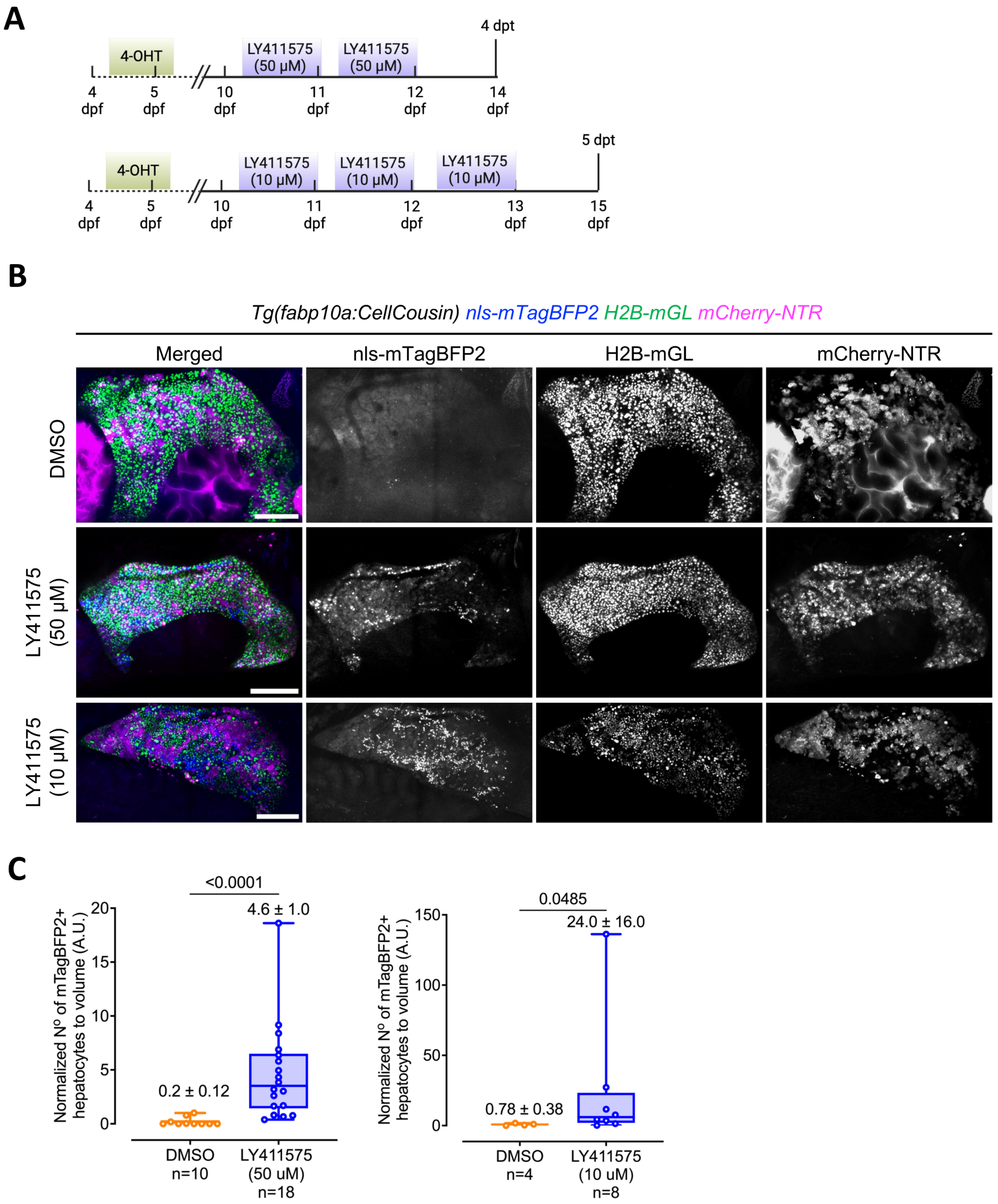

Supplementary Figure 7: Inhibition of Notch signalling pathway induces spontaneous transdifferentiation even in the absence of ablation.

(A) Schematic of the experimental strategy for inhibition of Notch signalling pathway in *Tg(fabp10a:CellCousin*) zebrafish larvae. Larvae were treated with 10 μM 4-OHT at 4.5 dpf for 24 hours. To inhibit the Notch pathway, larvae were treated with LY411575 (10 μM or 50 μM) for 16 hours at 3- and 2-days intervals, respectively, and then

analysed at 2 days post-treatment (dpt). **(B)** Confocal images of the liver in 15 dpf larvae treated with DMSO, 10 μM, or 50 μM LY411575 reveal the emergence of mTagBFP2+ *de novo* hepatocytes (blue) after LY411575 treatment suggesting spontaneous transdifferentiation from non-hepatocyte sources. Scale bars: 200μm. **(C)** Min-to-max box plot showing quantification of normalized mTagBFP2+ *de novo* hepatocytes shows a significant increase following treatment with 10 μM (left, n=8 animals) or 50 μM (right, n=18 animals) LY411575 compared to DMSO controls (n=4 animals and n=10 animals, respectively). Each dot represents one animal; mean ± SEM is indicated above each box. Statistical significance was assessed using the Mann–Whitney test.

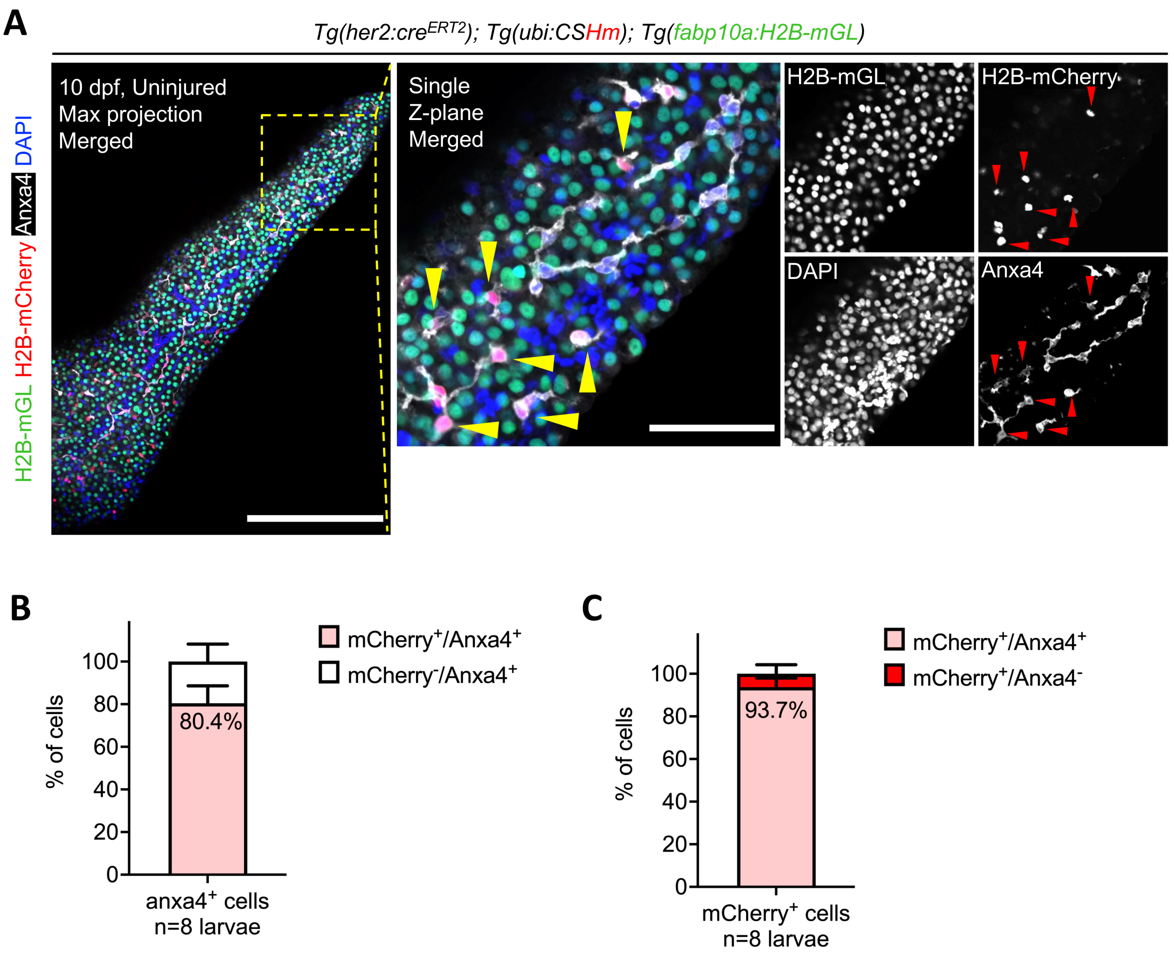

Supplementary Figure 8: her2+ cell lineage mainly contains cholangiocytes.

(A) The her2 lineage is marked with nuclear mCherry following 4-OHT treatment from 48 to 78 hpf, while hepatocytes are labelled with H2B-mGL. At 10 dpf, mCherry cells, but none of the H2B-mGL cells, exhibit Anxa4-immunofluorescence (grey). Anxa4 labelling is specific to cholangiocytes. Representative mCherry cells with Anxa4 label are marked with arrowheads. Scale bars: 200 μm, 50 μm (inset). **(B-C)** Barplot showing Mean+SD with number of animals listed below. **(B)** Quantification of the percentage of Anxa4+ (cholangiocytes) that are mCherry+ (her2 cell lineage). **(C)** Quantification of the percentage of mCherry+ (her2 cell lineage) that are Anxa4+ (cholangiocytes).

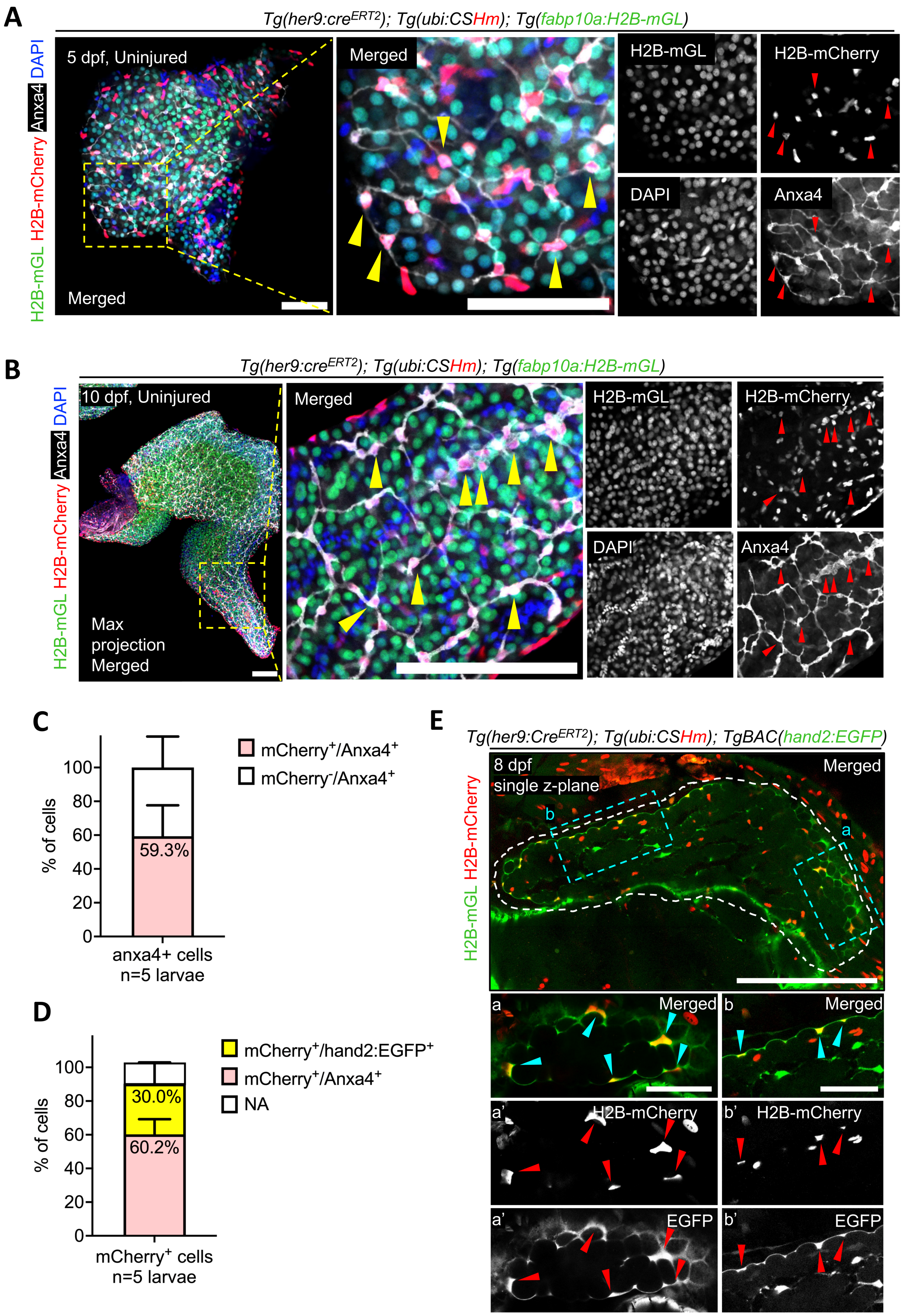
A
Tg(her9:creERT2); Tg(ubi:CSHm); Tg(fabp10a:H2B-mGL)
H2B-mGL H2B-mCherry Anxa4 DAPI
5 dpf, Uninjured
Merged
Merged
H2B-mGL
H2B-mCherry
DAPI
Anxa4
B
Tg(her9:creERT2); Tg(ubi:CSHm); Tg(fabp10a:H2B-mGL)
H2B-mGL H2B-mCherry Anxa4 DAPI
10 dpf, Uninjured
Max projection Merged
Merged
H2B-mGL
H2B-mCherry
DAPI
Anxa4
C
% of cells
100
80
60
40
20
0
59.3%
anxa4+ cells
n=5 larvae
mCherry+/Anxa4+
mCherry-/Anxa4+
D
% of cells
100
80
60
40
20
0
30.0%
60.2%
mCherry+ cells
n=5 larvae
mCherry+/hand2:EGFP+
mCherry+/Anxa4+
NA
E
Tg(her9:CreERT2); Tg(ubi:CSHm); TgBAC(hand2:EGFP)
H2B-mGL H2B-mCherry
8 dpf
single z-plane
Merged
a
b
Merged
Merged
a'
H2B-mCherry
b'
H2B-mCherry
a'
EGFP
b'
EGFP

Supplementary Figure 9: her9+ cell lineage contains cholangiocytes and hand2+ cells, but not hepatocytes.

(A, B) The her9 lineage is marked with nuclear mCherry following 4-OHT treatment from 48 to 78 hpf, while hepatocytes are labelled with H2B-mGL. At 5 dpf (A) and at 10 dpf (B), a subset of mCherry cells, but none of the H2B-mGL cells, exhibit Anxa4-immunofluorescence (grey). Anxa4 labelling is specific to cholangiocytes. Representative mCherry cells with Anxa4 label are marked with arrowheads. Scale bars: 50 μm (A), 100 μm (B). **(C-D)** Barplot showing Mean+SD with number of animals listed below. **(C)** Quantification of the percentage of Anxa4+ (cholangiocytes) that are mCherry+ (her9 cell lineage). **(D)** Quantification of the percentage of mCherry+ (her9 cell lineage) that are Anxa4+ (cholangiocytes) or *hand2*+. **(E)** Confocal image showing the overlap between her9-cell lineage (mCherry+ cells) and *hand2*+ cells (EGFP+ cells), marked with arrowhead. Scale bars: 200 μm, 50 μm (inset).

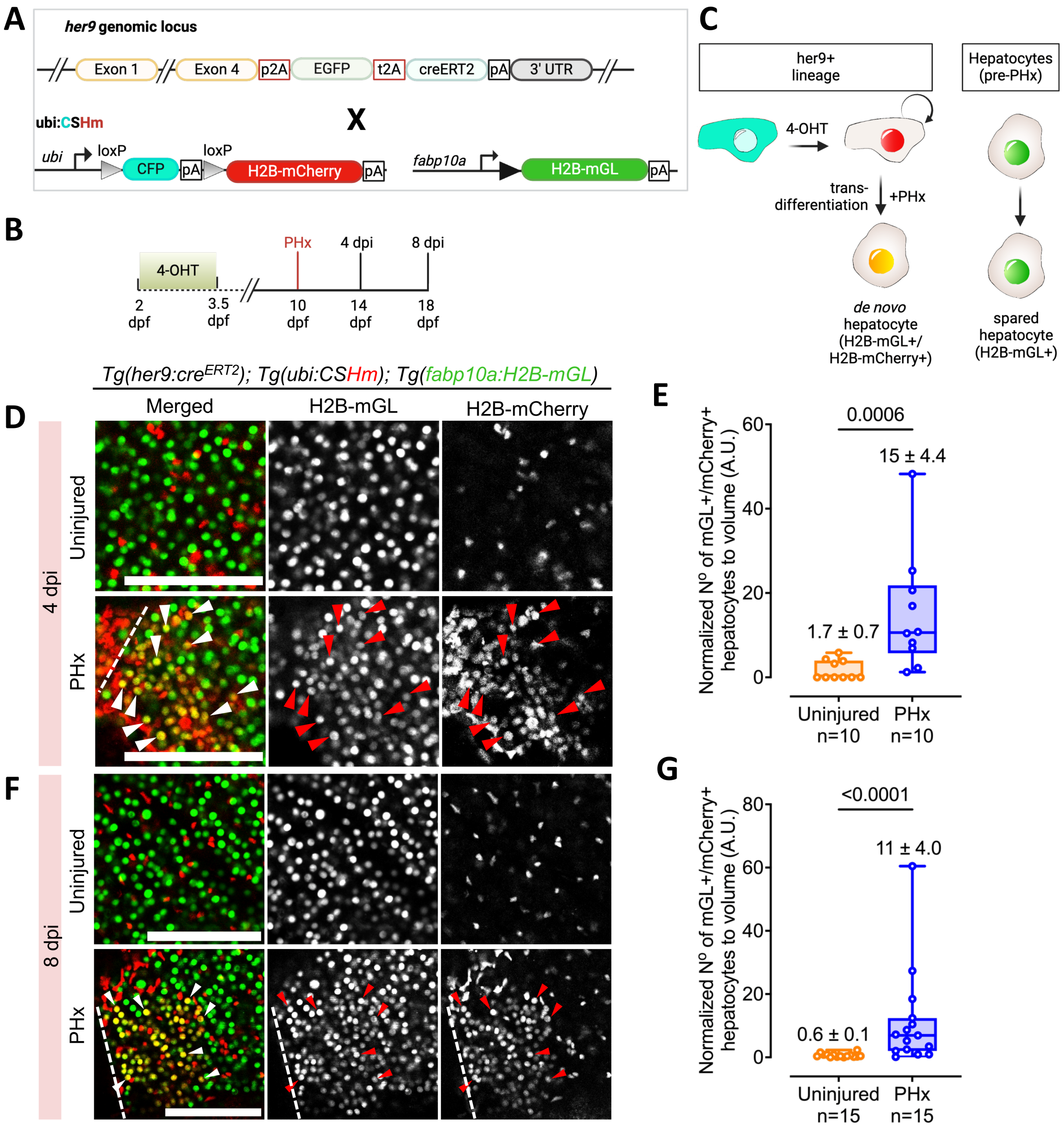

Supplementary Figure 10: Lineage tracing strategy to demonstrate the contribution of her9+ cells lineage to *de novo* hepatocytes.

(A) The Cre/loxP strategy used to trace her9-derived *de novo* hepatocytes. Fish carrying a knock-in of p2A-*EGFP-t2A-CreER^{T2}* in the her9 locus are crossed to a ubiquitous Cre-reporter line and a hepatocyte reporter line. **(B)** 4-OHT was administered from 48 hpf to 78 hpf, and the her9 lineage was traced at 4 and 8 dpi following PHx performed at 10 dpf. **(C)** Schematic illustrating the tracing of her9

lineage before and after PHx. her9-expressing cells are labelled with mCherry following 4-OHT treatment. After transdifferentiation into hepatocytes, these cells become H2B-mGL+/mCherry+ as hepatocytes are marked with H2B-mGL. **(D, F)** Confocal images of left lobe in uninjured and PHx conditions at 4 dpi (D) and at 8 dpi (F). Representative H2B-mGL+/mCherry+ hepatocytes are marked with arrowheads and resection site are shown with dashline. Scale bars: 100 μm. **(E, G)** Min-to-max boxplot showing quantification of the normalized number of H2B-mGL+/mCherry+ cells representing her9-derived *de novo* hepatocytes at 4 dpi (E) and at 8 dpi (G) shows a significant increase in animals subjected to PHx (n=10 animals, 10 animals, respectively) compared to uninjured animals (n=10 animals, 15 animals, respectively) Each dot represents one animal; mean ± SEM is indicated above each box. Statistical significance was assessed using the Mann–Whitney test.

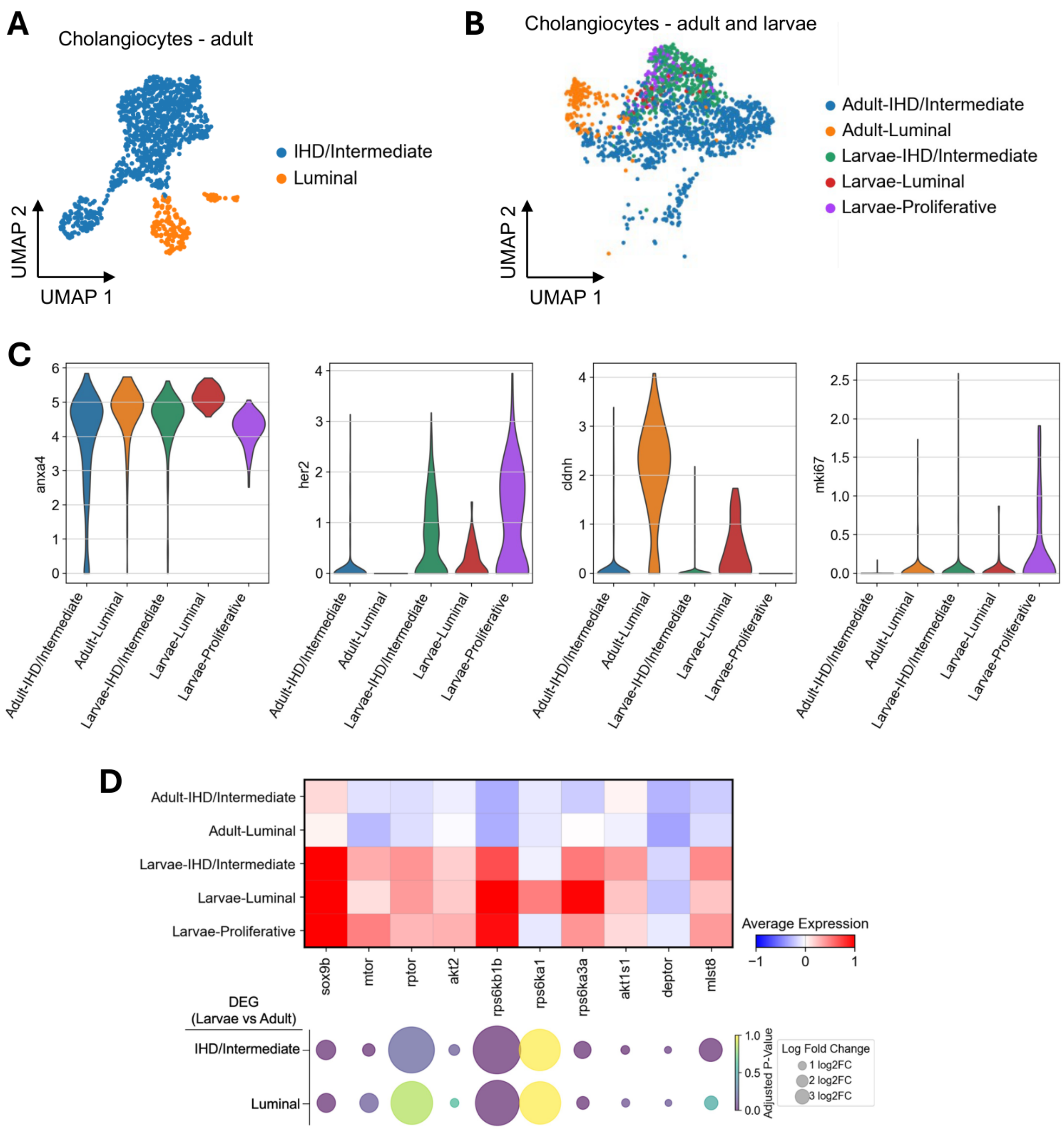

Supplementary Figure 11: Comparative transcriptomic analysis of cholangiocyte subclusters from larval and adult zebrafish livers.

(A) UMAP visualization of cholangiocytes from uninjured 18 mpf adult zebrafish liver, showing subclustering into intrahepatic duct (IHD)/intermediate (blue) and luminal (orange) populations. **(B)** Integrated UMAP plot of cholangiocytes from 13 dpf larval and adult livers, color-coded by subcluster and developmental stage: Adult-IHD/Intermediate (blue), Adult-Luminal (orange), Larval-IHD/Intermediate (green),

Larval-Luminal (red), and Larval-Proliferative (purple). **(C)** Violin plots showing expression of representative marker genes across cholangiocyte subclusters from larval and adult livers, including *anxa4* (general cholangiocyte), *her2* (IHD/intermediate), *cdh1* (luminal), and *mki67* (proliferative). **(D)** Top: Heatmap showing average expression of selected genes associated with mTORC1 signaling and cholangiocyte plasticity across each cholangiocyte subcluster. Bottom: Bubble plot showing differentially expressed genes (DEGs) related to the mTORC1 pathway between larval and adult cholangiocytes. Dot size represents the adjusted p-value, and color intensity reflects log2 fold change.

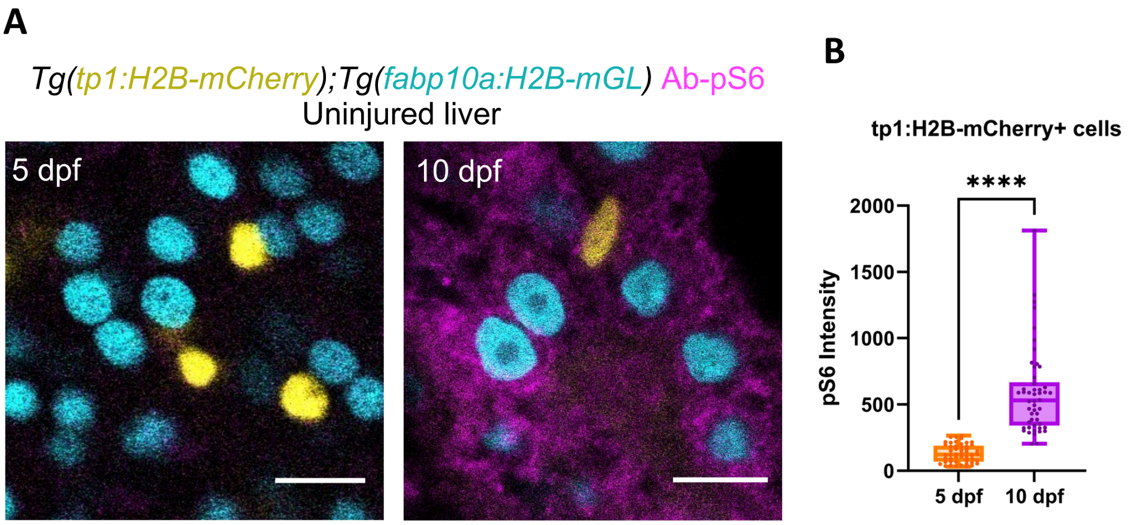

Supplementary Figure 12: mTORC1 signalling differs between early and late larval stages.

(A) Confocal images of livers from 5 dpf and 10 dpf *Tg(tp1:H2B-mCherry); Tg(fabp10a:H2B-mGL)* stained with pS6 antibody. **(B)** Comparison of pS6 signal intensity in mCherry+ cells (tp1+ cholangiocytes) at 5 dpf and 10 dpf. Data are represented as min-to-max box plots (n = 50 cells per condition). Statistical significance was assessed using the Mann–Whitney test (**** $p < 0.0001$). Scale bars: 10 μm.
