# Supplementary material for: Cholangiocytes contribute to hepatocyte regeneration after partial liver injury during growth spurt in zebrafish
Source: Nat Commun. 2025 Jun 6;16:5260. doi: 10.1038/s41467-025-60334-y (PMC12144294; doi:10.1038/s41467-025-60334-y)
Supplement: Supplementary file 2 — Description of Additional Supplementary Information [file 41467_2025_60334_MOESM2_ESM.docx]

**Description of Additional Supplementary Files**

File Name: Supplementary Data 1

Description: *Differentially expressed genes between cholangiocytes from larval and adult zebrafish livers.* Table showing gene name, log2FoldChange and False Discovery Rate (FDR) values.

File Name: Supplementary Data 2

Description: *Differentially expressed genes between IHD/intermediate cholangiocytes from larval and adult zebrafish livers.* Table showing gene name, log2FoldChange and False Discovery Rate (FDR) values.

File Name: Supplementary Data 3

Description: *Differentially expressed genes between luminal cholangiocytes from larval and adult zebrafish livers.* Table showing gene name, log2FoldChange and False Discovery Rate (FDR) values.

File Name: Supplementary Movie 1

Description: *Proliferative cells localize close to the edge of the regenerate at 1 dpi after PHx.* A whole mount image of Tg(fabp10:H2B-mGreenLantern); Tg(tp1:H2B-mCherry) stained with EdU to label proliferating cells at 1 dpi after PHx.
