# Supplementary material for: Cholangiocytes contribute to hepatocyte regeneration after partial liver injury during growth spurt in zebrafish
Source: Nat Commun. 2025 Jun 6;16:5260. doi: 10.1038/s41467-025-60334-y (PMC12144294; doi:10.1038/s41467-025-60334-y)
Supplement: Supplementary file 7 — Reporting Summary [file 41467_2025_60334_MOESM7_ESM.pdf]

nature portfolio

Corresponding author(s): Sumeet Pal Singh

Last updated by author(s): May 14, 2025

Reporting Summary

Nature Portfolio wishes to improve the reproducibility of the work that we publish. This form provides structure for consistency and transparency in reporting. For further information on Nature Portfolio policies, see our Editorial Policies and the Editorial Policy Checklist.

Statistics

For all statistical analyses, confirm that the following items are present in the figure legend, table legend, main text, or Methods section.

n/a	Confirmed	
☐	☒	The exact sample size (*n*) for each experimental group/condition, given as a discrete number and unit of measurement
☐	☒	A statement on whether measurements were taken from distinct samples or whether the same sample was measured repeatedly
☐	☒	The statistical test(s) used AND whether they are one- or two-sided<br>*Only common tests should be described solely by name; describe more complex techniques in the Methods section.*
☒	☐	A description of all covariates tested
☐	☒	A description of any assumptions or corrections, such as tests of normality and adjustment for multiple comparisons
☐	☒	A full description of the statistical parameters including central tendency (e.g. means) or other basic estimates (e.g. regression coefficient) AND variation (e.g. standard deviation) or associated estimates of uncertainty (e.g. confidence intervals)
☐	☒	For null hypothesis testing, the test statistic (e.g. *F*, *t*, *r*) with confidence intervals, effect sizes, degrees of freedom and *P* value noted<br>*Give P values as exact values whenever suitable.*
☒	☐	For Bayesian analysis, information on the choice of priors and Markov chain Monte Carlo settings
☒	☐	For hierarchical and complex designs, identification of the appropriate level for tests and full reporting of outcomes
☒	☐	Estimates of effect sizes (e.g. Cohen's *d*, Pearson's *r*), indicating how they were calculated

Our web collection on statistics for biologists contains articles on many of the points above.

Software and code

Policy information about availability of computer code

Data collection	No custom code used in this manuscript.
Data analysis	Single-cell RNA-Sequencing analysis was performed using Scanpy (v1.10.2). Pseudotemporal analysis was performed using Monocle3. Cell counting was performed using Imaris 10.0 and Fiji (Image J), version 2.16.0. Statistical analysis was assessed using Prism software 9 version 9.3.1 (GraphPad Software, Inc, La Jolla, CA, USA).

For manuscripts utilizing custom algorithms or software that are central to the research but not yet described in published literature, software must be made available to editors and reviewers. We strongly encourage code deposition in a community repository (e.g. GitHub). See the Nature Portfolio guidelines for submitting code & software for further information.

Data

Policy information about availability of data

All manuscripts must include a data availability statement. This statement should provide the following information, where applicable:

- Accession codes, unique identifiers, or web links for publicly available datasets
- A description of any restrictions on data availability
- For clinical datasets or third party data, please ensure that the statement adheres to our policy

The raw files and raw count table from deep sequencing can be accessed at Gene Expression Omnibus (GEO). Plasmids construct in the manuscript have been deposited to Addgene. Raw images and image analysis pipeline are available upon request to the corresponding author.

Research involving human participants, their data, or biological material

Policy information about studies with human participants or human data. See also policy information about sex, gender (identity/presentation), and sexual orientation and race, ethnicity and racism.

Reporting on sex and gender	N/A
Reporting on race, ethnicity, or other socially relevant groupings	N/A
Population characteristics	N/A
Recruitment	N/A
Ethics oversight	N/A

Note that full information on the approval of the study protocol must also be provided in the manuscript.

Field-specific reporting

Please select the one below that is the best fit for your research. If you are not sure, read the appropriate sections before making your selection.

- [x] Life sciences
- [ ] Behavioural & social sciences
- [ ] Ecological, evolutionary & environmental sciences

For a reference copy of the document with all sections, see nature.com/documents/nr-reporting-summary-flat.pdf

Life sciences study design

All studies must disclose on these points even when the disclosure is negative.

Sample size	We have estimated the number of samples to ensure a power of 80% with a statistical significance level of 5% using R. In addition, we designed our experiments based on previous experience.
Data exclusions	No data was excluded from analysis.
Replication	Results have been replicated in at least 2 independent experiments
Randomization	Animals were selected at random for experimentation.
Blinding	No blinding was performed.

Reporting for specific materials, systems and methods

We require information from authors about some types of materials, experimental systems and methods used in many studies. Here, indicate whether each material, system or method listed is relevant to your study. If you are not sure if a list item applies to your research, read the appropriate section before selecting a response.

Materials & experimental systems

n/a	Involved in the study	
☐	☒	Antibodies
☒	☐	Eukaryotic cell lines
☒	☐	Palaeontology and archaeology
☐	☒	Animals and other organisms
☒	☐	Clinical data
☒	☐	Dual use research of concern
☒	☐	Plants

Methods

n/a	Involved in the study	
☒	☐	ChIP-seq
☐	☒	Flow cytometry
☒	☐	MRI-based neuroimaging

Antibodies

Antibodies used	Primary antibodies: mouse anti-Anxa4/2F11 (1:100, Abcam, ab71286), anti-pS6 (1:200, CST, 2215S), rabbit anti-mCherry (1:1000, CST, 43590). Secondary antibodies (Alexa Fluor 647 donkey anti-mouse, Cy3 donkey anti-rabbit).
Validation	The antibodies have been previously used in literature.

Animals and other research organisms

Policy information about studies involving animals; ARRIVE guidelines recommended for reporting animal research, and Sex and Gender in Research

Laboratory animals	Danio rerio (Zebrafish)
Wild animals	N/A
Reporting on sex	For studies in larvae, sex determination is not possible. For adults, the experiment was performed in male and females separately.
Field-collected samples	N/A
Ethics oversight	Ethical Committee for Animal Welfare (CEBEA) from the Université Libre de Bruxelles.

Note that full information on the approval of the study protocol must also be provided in the manuscript.

Plants

Seed stocks	N/A
Novel plant genotypes	N/A
Authentication	N/A

Flow Cytometry

Plots

Confirm that:

- [ ] The axis labels state the marker and fluorochrome used (e.g. CD4-FITC).
- [ ] The axis scales are clearly visible. Include numbers along axes only for bottom left plot of group (a 'group' is an analysis of identical markers).
- [ ] All plots are contour plots with outliers or pseudocolor plots.
- [x] A numerical value for number of cells or percentage (with statistics) is provided.

Methodology

Sample preparation	The liver was dissociated into single cells by incubation it in TrypLE (Thermo Fisher, 12563029) at 37°C in a benchtop shaker set at 1000 rpm for 15 min. Following dissociation, TrypLE was inactivated with goat serum. To remove undissociated chunks and debris, the solution was passed through a 40m cell strainer (Miltenyi Biotec, 130-041-407). Cells were pelleted by centrifugation at 500 g for 5 min at 4°C with soft-stop setting. The supernatant was carefully discarded and the pellet re-suspended in 500l of PBS. To remove dead cells, Calcein violet (Thermo Fisher, C34858) or Draq7 (Thermo Fisher, D15105) was added at a final concentration of 1 µM and 3 µM, respectively, and the cell suspension was incubated at room temperature for 20 min. The single-cell preparation was sorted using appropriate gates, including excitation with UV (405 nm) or a 633 nm laser for identifying live cells with Calcein+ or Draq7-, respectively. FACS was performed using a 100 µm nozzle. Sorting time did not exceed 15 min.
Instrument	FACS-Aria II (BD Bioscience)
Software	FCSalyzer Version 0.9.22-alpha (https://sourceforge.net/projects/fcsalyzer/)
Cell population abundance	Cell abundance ranged from 0 % - 20 %.
Gating strategy	Cells were sorted based on fluorescence intensity. A negative control of liver from wild-type animal (with no fluorescence) was used to make the gates. (The gating strategy is provided in the Source Data file: Panel 4E).

- [x] Tick this box to confirm that a figure exemplifying the gating strategy is provided in the Supplementary Information.
